# Neurological outcomes of extended thymectomy for thymomatous myasthenia gravis: Subxiphoid vs. trans-sternal approaches

**DOI:** 10.3389/fsurg.2022.973954

**Published:** 2022-09-06

**Authors:** Haoshuai Zhu, Zhihao Liu, Xiaojing Yao, Jianyong Zou, Bo Zeng, Xin Zhang, Zhenguang Chen, Chunhua Su

**Affiliations:** The Thoracic Surgery Department of The First Affiliated Hospital of Sun Yat-sen University, Guangzhou, China

**Keywords:** thymoma, myasthenia gravis, subxiphoid approach, quantitative myasthenia gravis score, neurological outcome

## Abstract

**Background:**

The subxiphoid approach has been widely used recently. However, there is little data focusing on neurological outcomes in patients with thymomatous myasthenia gravis (MG) who underwent subxiphoid thoracoscopic thymectomy. The purpose of this study was to compare the neurological outcomes of patients with thymomatous MG who underwent extended thymectomy with a subxiphoid or transthoracic approach 1 year postoperatively.

**Methods:**

The records of patients with Masaoka stage I and II thymomas who underwent extended thymectomy from January 2019 to December 2020 with tumor size less than 5 cm and thymomatous MG were retrospectively reviewed and evaluated. Neurological outcomes were measured by a quantitative myasthenia gravis score (QMGS), with a 2.3-point reduction in QMGS associated with improvement in clinical MG status. The clinical efficacy and variables affecting the outcomes were assessed using the Kaplan–Meier method and Cox proportional hazard regression analysis.

**Results:**

A total of 89 patients were included in the analysis, of which 44 had a subxiphoid approach and 45 had a trans-sternal approach. Mean QMGS decreased from 12 at initial diagnosis to 8.7 preoperatively and 5.6 at 12 months postoperatively in the subxiphoid group and from 12.1 to 8.9 to 6.0 in the transthoracic group. Thirteen patients (28.9%) who underwent the trans-sternal approach and 10 (22.7%) who underwent the subxiphoid approach did not have an improved clinical status compared with their preoperative status. The median time to clinical improvement was 3 months (95% CI, 2.15–3.85) for the subxiphoid approach and 6 months (95% CI, 5.54–6.46) for the trans-sternal approach. Univariate results showed that the subxiphoid approach was associated with a faster improvement in clinical status (HR = 1.701, 95% CI, 1.044–2.773, *P *< 0.05), and age ≦48 was associated with a faster improvement in clinical status (HR = 1.709, 95% CI, 1.044–2.799, *P *< 0.05). The multivariate model including age ≦48 (HR = 1.837, 95% CI, 1.093–3.086, *P *= 0.022) and the subxiphoid approach (HR = 1.892, 95% CI, 1.127–3.177, *P *= 0.016) was significantly associated with a faster improvement in clinical status.

**Conclusions:**

In patients with Masaoka stage I and II thymoma who underwent thymectomy, with tumor size less than 5 cm and thymomatous MG, age ≦48 years and the subxiphoid approach were associated with a rapid improvement in clinical status.

## Introduction

Extended thymectomy has been the mainstay of treatment for thymoma combined with myasthenia gravis (MG) (1, 2). The traditional method of surgical treatment is median sternotomy (3). Recently, minimally invasive techniques, including thoracoscopic and robot-assisted thoracic surgery, have become the standard of care for early-stage thymic tumors (4–6). However, the appropriate surgical approach remains controversial due to the recommendation to remove all thymus glands, both encapsulated and extracapsular tissues, during surgery, resulting in a better outcome for patients with MG. Several studies have shown that minimally invasive thymectomy can reduce intraoperative blood loss, postoperative pain, and postoperative complications (7–9). However, the neurological outcomes of MG after minimally invasive thymectomy are debatable. Few studies have focused on whether minimally invasive surgery is effective in improving neurological outcomes in patients with MG-affected thymoma. Franca Melfi suggested that robotic surgery for patients with thymoma and concomitant MG could be effective in improving neurological prognosis (10).

The subxiphoid approach has been used successfully in many centers in the last decade. Our center has been using the subxiphoid and subcostal arch approaches since 2017. In contrast to the unilateral approach, which makes it challenging to visualize the contralateral phrenic clearly, the subxiphoid approach allows adequate visualization of the entire anterior mediastinal space (11, 12). Furthermore, it was reported that subxiphoid and subcostal arch thoracoscopic thymectomy appeared to be a safe and feasible procedure for early-stage thymoma and MG (10, 13). However, they did not explore the neurological outcomes of MG under this approach.

QMGS may be more sensitive than other methods in detecting differences in neurological outcomes in MG (14, 15). A randomized trial comparing thymectomy plus prednisone with prednisone alone for MG used QMGS to assess outcomes and reported improved clinical outcomes for thymectomy compared with those for prednisone alone for 3 years. The results of the trial showed that a 2.3-point reduction in QMGS was associated with an improvement in the clinical status of MG, and the time to achieve a 2.3-point reduction was approximately 3 months (1).

A retrospective study was conducted to evaluate the clinical and neurological outcomes of extended thymectomy under video-assisted thoracoscopic surgery (VATS) through the subxiphoid and subcostal arches and compared with median sternotomy in patients diagnosed with early phrenic thymoma with thymomatous MG.

## Materials and methods

### Patients and treatments

This study was approved by the Clinical Research Committee of the First Affiliated Hospital of Sun Yat-sen University. Because of the retrospective nature of the study, the requirement for informed patient consent was waived. At our hospital, a team of experienced neurologists and thoracic surgeons worked together to treat patients diagnosed with thymoma and thymomatous MG.

Clinical data were collected from January 2019 to December 2020 from all consecutive patients with Masaoka stage I and II thymoma and thymomatous MG who underwent thoracoscopic thymectomy *via* subxiphoid and subcostal arch or open sternotomy. The diagnosis of thymoma was based on computed tomography (CT) imaging of the chest. Tumors less than 5 cm in size with well-defined borders were considered suitable for the VATS approach under the subxiphoid and subcostal arches. The staging of thymoma was evaluated according to Masaoka's staging system (16). Masaoka stage I and II thymomas with tumor size less than 5 cm were collected. The severity of MG was assessed according to the clinical classification of the Myasthenia Gravis Foundation of America (MGFA) (17). Before surgery, MG was initially treated to control symptoms, as decided by neurologists. Thymectomy was performed when MG symptoms improved significantly in MGFA classes less than IIB, and the daily dosage of prednisone was <20 mg/day.

All patients who underwent surgery were evaluated by thoracic surgeons, neurologists, and anesthetists prior to surgery. Informed consent was obtained from all patients before surgery. A routine preoperative evaluation was performed to rule out distant metastases and evaluate cardiac and pulmonary functions. Extensive thymectomy was performed by thoracic surgeons by open sternotomy or under VATS using subxiphoid and subcostal arch approaches.

All surgical specimens were subjected to histopathological examination. Thymomas were classified according to the new World Health Organization classification. The pathological staging of thymomas was performed according to the Masaoka staging system. Only patients with thymoma confirmed by histopathological examination and not type C (thymic carcinoma) were included in the analysis. The patients were seen in the neurology clinic at 1, 3, 6, and 12 months postoperatively to evaluate their neurological status using QMG scores, which were recorded at the time of preoperative confirmation of the initial diagnosis and the day before surgery. In this study, the QMG score was used as an outcome measure, which was also used in our study. The study showed that a 2.3-point reduction in score was associated with an improvement in clinical status. The doses of medications were gradually reduced according to the improvement in the symptoms of MG. Only patients with at least 1 year of follow-up and complete follow-up data for neurological outcomes were included in the analysis.

All patients were supine on the operating table with the surgeon standing between their legs. The assistant was on the right side of the patient. A 3-cm incision was made at the lower edge of the xiphoid to set up the thoracoscope. The rectus abdominis muscle was dissected, and the posterior sternum space was created by finger dissection. Under the guidance of the operator's finger, two 5 mm extrapleural thoracic ports were created at the intersection of the midclavicular line and bilateral costal arches to introduce thoracoscopic grasping forceps and a harmonic scalpel. A 30° oblique 10-mm thoracoscope was introduced through a subxiphoid incision. A pneumomediastinum was created by an 8-cm H_2_O positive pressure carbon dioxide (CO_2_) insufflation to enlarge the retrosternal space and facilitate tumor dissection. During the operation, both the right and the left mediastinal pleura were opened. The entire thymus and thymoma and all associated adipose tissue were excised en-bloc. The prepericardial fat and the fat pads near the cardiac–diaphragmatic angles, the aortopulmonary window, and the lower poles of the thyroid gland were carefully dissected and excised. The superior vena cava, both the innominate veins and the aorta, were skeletonized. All samples were placed in a plastic bag and removed from the mediastinum through the subxiphoid port. An 18-F drainage tube was inserted into the mediastinum through the left costal arch port (13, 18). Patients with converted open sternotomy intraoperative were excluded from the analysis.

Demographic data, as well as intraoperative and postoperative results, were collected and analyzed. A formal pain assessment for the study was also performed using visual analog scales at 24 and 72 h after surgery. The purpose of this study was to compare the neurological outcomes of the two approaches 1 year after the operation.

### Statistical analysis

Continuous variables were reported as mean ± standard deviation (SD) and median (range), whereas categorical variables were presented as numbers and percentages. To investigate the association between independent variables and improved clinical status, univariate and multivariate Cox proportional hazards regression models were used. Significant variables in the univariate results were entered into the multivariate model, and significant variables in the multivariate results were recognized as factors associated with the improvement in clinical status. Associated factors were further used as group factors for Kaplan–Meier survival analysis and the log-rank test to observe changes in the rates of achieving clinical status improvement during postoperative follow-up. All analyses were performed using IBM SPSS version 25 software (SPSS Statistics V25, IBM Corporation, Somers, NY, USA). In all analyses, a two-tailed value of *P* < 0.05 was considered to indicate statistical significance.

## Results

**Figure 1:**
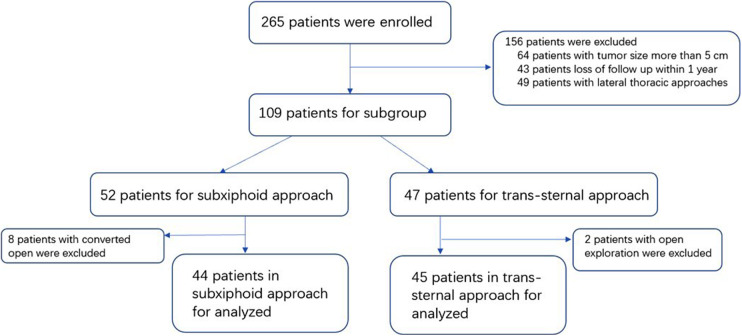
Flowchart of patient disposition.

In total, 265 consecutive patients were enrolled from January 2019 to December 2020. As shown in Figure 1, 89 patients were included in the analysis, 44 of whom had a subxiphoid approach, and 45 had a trans-sternal approach. The mean age of these 89 patients was 48.8 years. The characteristics of the clinical and pathological data between the subxiphoid approach and the trans-sternal approach groups are given in Table 1. There were no statistical differences between the two groups in terms of gender, age, MGFA clinical classification, WHO histological type, maximum tumor size, and Masaoka–Koga stage. Pain scores were significantly higher at 24 and 72 h after surgery in patients with the trans-sternal approach. The duration of operation and blood loss differed significantly between the two approaches, and we did not compare these two variables in Table 1. However, the mean operative duration for the subxiphoid approach was 98 min, and the mean blood loss was 29.2 ml.

**Table 1:** Characteristics of clinical and pathological data between subxiphoid approach and trans-sternal approach groups.

Clinical characterstics	Subxiphoid group	Trans-sternal group	*P*
Gender, *n*			0.519
Male	28	25	
Female	16	20	
Age (years), median (range)	49.1 (18–70)	48.4 (25–76)	0.785
MGFA classification, *n*			0.937
I	2	2	
IIA	4	6	
IIB	20	20	
III	18	17	
WHO histologic type, *n*			0.784
A/AB	7	9	
B1/B2/B3	37	36	
Maximal tumor size, mean (cm)	2.9 ± 0.7	3.1 ± 0.7	0.162
Masaoka–Koga stage, *n*			0.714
I	41	40	
II	3	5	
Bleeding, mean (ml)	29.2 ± 10.7	151.1 ± 39.2	<0.001
Postoperative pain [0–10 (VAS score)]			
24 h postoperatively	4.1 ± 1.1	6.5 ± 0.8	<0.001
72 h postoperatively	2.1 ± 0.7	3.7 ± 1.0	<0.001

MGFA, Myasthenia Gravis Foundation of America.

### Neurological outcomes

The comparative data on neurological outcomes between the subxiphoid and the trans-sternal approach groups are presented in Table 2. There were no statistical differences between the two groups in terms of preoperative administration of pyridostigmine, steroid administration, immunosuppressive medications, or perioperative immunoglobulin infusion. There were two cases of postoperative MG crisis in the subxiphoid group and four cases in the trans-sternal group.

**Table 2:** Comparison of neurological outcomes between subxiphoid approach and trans-sternal approach groups.

Variables	Subxiphoid group	Trans-sternal group	*P*
Pyridostigmine daily preoperatively, mean (mg)	194.3 ± 31.4	191.3 ± 29.5	0.645
Steroids preoperatively (yes/no)	13/31	14/31	1
Immunosuppressive drugs preoperatively (yes/no)	8/36	8/37	1
Perioperative immunoglobin (yes/no)	18/26	14/31	0.382
Postoperative MG crisis, *n*	2	4	0.677
QMGs at initial diagnosis	12.0 ± 2.8	12.1 ± 2.5	0.909
QMGs preoperatively	8.7 ± 1.4	8.9 ± 1.2	0.514
QMGs 1 month postoperatively	6.7 ± 1.4	8.6 ± 1.6	<0.001
QMGs 3 months postoperatively	6.3 ± 1.7	7.2 ± 1.7	0.011
QMGs 6 months postoperatively	6.0 ± 1.6	6.4 ± 1.7	0.217
QMGs 12 months postoperatively	5.6 ± 1.5	6.0 ± 1.7	0.317

QMG, quantitative myasthenia gravis score; MG, myasthenia gravis.

**Figure 2:**
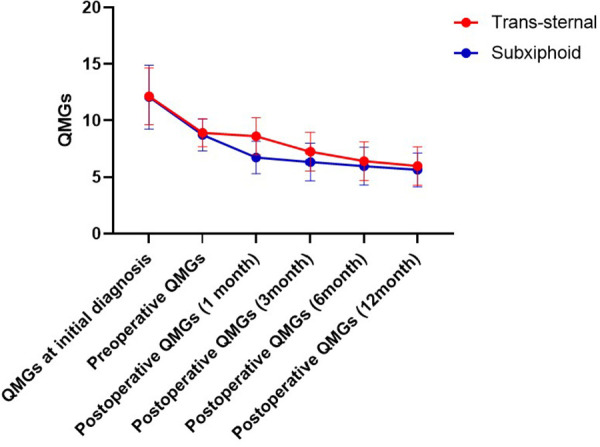
Change of QMGs from the initial diagnosis to 12 months follow-up. QMG, quantitative myasthenia gravis score.

The changes in QMG during the 12-month postoperative follow-up are shown in Figure 2. As shown in Table 2, the mean QMG decreased from 12 at initial diagnosis to 8.7 preoperatively and 5.6 at 12 months postoperatively in the subxiphoid group and from 12.1 to 8.9 and 6.0 in the trans-sternal group. There were no statistical differences in mean QMGS between the two groups at initial diagnosis, preoperative time, 6 months postoperatively, and 12 months postoperatively. Mean QMGS in the subxiphoid group was significantly lower at 1 month and 3 months postoperatively. The curves for the two groups separate only at 1 and 3 months postoperatively but coincide at 6 months postoperatively.

To investigate the impact of the surgical approach on postoperative neurological outcomes, a 2.3-point reduction in the QMG score prior to surgery was associated with an improvement in clinical status. Kaplan–Meier analysis indicated that the median time to achieve improved clinical status was 3 months (95% CI, 2.15–3.85) for the subxiphoid approach and 6 months (95% CI, 5.54–6.46) for the trans-sternal approach. Thirteen patients (28.9%) underwent the trans-sternal approach, and 10 (22.7%) who underwent the subxiphoid approach did not improve their clinical status compared with the preoperative period.

Univariate and multivariate analyses were used to explore the independent variables associated with a faster improvement in clinical status. The results are given in Table 3. Since the event was defined as a reduction of 2.3 QMGS, which implies an improvement in clinical status, an estimated hazard ratio (HR) <1 indicates a slower improvement in clinical status, whereas a HR >1 indicates a faster improvement in clinical status. Univariate results showed that the subxiphoid approach was associated with a faster improvement in clinical status (HR = 1.701, 95% CI, 1.044–2.773, *P *< 0.05), whereas age ≦48 was associated with a faster improvement in clinical status (HR = 1.709, 95% CI, 1.044–2.799, *P *< 0.05). Age ≦48 (HR = 1.837, 95% CI, 1.093–3.086, *P *= 0.022) and the subxiphoid approach (HR = 1.892, 95% CI, 1.127–3.177, *P *= 0.016) remained significant in the multivariate model.

**Table 3:** Univariate and multivariate Cox regression analyses of improved clinical status.

Variables	Univariate		Multivariate	
	HR (95% CI)	*P*	HR (95% CI)	*P*
Gender		0.872		
Female	0.96 (0.588–1.57)			
Male	1			
Age		0.033		0.022
≦48	1.709 (1.044–2.799)		1.837 (1.093–3.086)	
≧49	1		1	
MGFA classification		0.723		
I + IIA	0.889 (0.465–1.70)			
IIB + III	1			
WHO histologic type		0.227		
A + AB	1.428 (0.801–2.543)			
B1 + B2 + B3	1			
Masaoka–Koga stage		0.901		
I	1.055 (0.455–2.444)			
II	1			
Steroids preoperatively		0.279		
Yes	0.736 (0.423–1.281)			
No	1			
Immunosuppressive drugs preoperatively		0.326		
Yes	0.702 (0.347–1.422)			
No	1			
Perioperative immunoglobin		0.339		
Yes	0.773 (0.457–1.31)			
No	1			
Surgical approach		0.033		0.016
Subxiphoid	1.701 (1.044–2.773)		1.892 (1.127–3.177)	
Trans-sternal	1		1	

MGFA, Myasthenia Gravis Foundation of America; HR, hazard ratio; CI, confidence interval.

## Discussion

Thoracoscopic subxiphoid and subcostal arch extended thymectomy is a feasible intervention for patients with early-stage thymoma and thymomatous MG (10, 13). For early-stage thymomas with a tumor size of less than 5 cm, the subxiphoid approach can provide adequate visualization of the entire anterior mediastinum space during operation. The use of this approach allows for extended thymectomy and adipose tissue dissection. According to the ITMIG guidelines, after identification of the right and left phrenic nerves, the anterior mediastinal adipose tissue must be removed to ensure oncological and neurological radicality, followed by dissection from the jugular to the anterior pericardiophrenic angle (19). Trans-sternal thymectomy has long represented the standard surgical approach for treating patients with thymoma and thymomatous MG with excellent visualization of the anterior mediastinum to ensure appropriate control of nerves and vessels (4). The trans-sternal approach was adopted as a control group to assess the neurological outcomes of thymomatous MG after extended thymectomy with a subxiphoid approach.

Several studies have confirmed the safety of the subxiphoid approach when applied to anterior mediastinal lesions (18, 20, 21). The approach *via* subxiphoid and subcostal arches was adopted in our center in 2017. During the early learning process, the procedure was converted to open sternotomy due to hemorrhage. After a 5–10 case learning curve, only patients with extensive invasion required conversion to an open sternotomy. The mean operative time in the subxiphoid group was 98 min, similar to other studies (13, 18, 21, 22) with a range of 95–147 min. The blood loss in our study was 29.2 ml, which was also similar to their results, with a range of 25.5–73.8 ml. The subxiphoid approach was less invasive and had a faster postoperative recovery. Most patients were discharged on the third or fourth postoperative day.

The postoperative myasthenia crisis after thymectomy was reported to be between 6% and 34% (23). However, this crisis was reported in the results of several studies (24). The causes of the myasthenia crisis were complicated. In the present study, we considered pain to play an important role in the postoperative myasthenia crisis. The subxiphoid group had lower VAS scores, contributing to less respiratory failure and better recovery. The postoperative myasthenia crisis was also less frequent in the subxiphoid group. Furthermore, a comparison of the perioperative and follow-up outcomes of patients with MG who underwent subxiphoid-subcostal or unilateral thoracoscopic thymectomy showed that only pain scores were significantly lower in the subxiphoid group postoperatively. However, the myasthenia crisis was less frequent, and the neurological results were better (21, 22).

It is well known that the course of MG is different in patients diagnosed with thymoma or benign disease (25). Studies have shown that thymoma is significantly associated with the inability to achieve complete stable remission (CSR) at long-term follow-up (26). CSR was not adopted as an evaluation indicator in this study due to the short follow-up period of 1 year. QMGS is a continuous variable and a favorable measure. Furthermore, there was an association between myasthenia symptoms and more aggressive forms of thymoma (27). However, the patients in this study were diagnosed with Masaoka stage I and II thymoma. The mean QMG decreased from 8.7 preoperatively to 5.6 at 12 months postoperatively in the subxiphoid group and from 8.9 to 6.0 in the trans-sternal group. Approximately 71.1% of the patients in the open group and 78.3% in the subxiphoid group had better neurological status. This is comparable to the results of other studies (22, 28, 29).

Patients in the subxiphoid group could see an early improvement in neurological status within 3 months, while those in the open group improved within 6 months postoperatively. The mean QMG score decreased rapidly from preoperative to postoperative, indicating a statistically significant improvement in clinical neurological status. However, the mean QMG scores were similar at 6 months, implying no statistically significant differences in long-term neurological outcomes between the two groups. Viet Anh Le reported that when they compared left-sided and right-sided VATS, as well as VATS and open surgery, there was no association between MG results and VATS sites between the two surgical approaches (30). In addition, another study showed no significant correlation between surgical approach and neurological outcomes (22). However, robotic surgical treatment for patients with thymoma and concomitant MG effectively improved neurological outcomes (10).

## Conclusion

In patients with Masaoka stage I and II thymoma who underwent thymectomy, with tumor size less than 5 cm and thymomatous MG, age 48 years and the subxiphoid approach were associated with a faster improvement in clinical status. Our previous study also showed that younger patients could have better neurological outcomes (31).

### Limitation

This monocentric retrospective study had several limitations. Due to the retrospective nature of the study, randomization was absent, and selection bias could not be eliminated. The number of patients and the duration of the follow-up period should be increased to confirm the neurological and oncological results. A prospective multicenter comparative study with other surgical techniques (unilateral thoracoscopic thymectomy) would be more appropriate.

## Data Availability

The raw data supporting the conclusions of this article will be made available by the authors, without undue reservation.
